# *Luticola edaphica* sp. nov. (Diadesmidaceae, Naviculales) from the Soil of the Russian Far East (Primorsky Territory, Russia)

**DOI:** 10.3390/plants15060897

**Published:** 2026-03-13

**Authors:** Veronika B. Bagmet, Arthur Yu. Nikulin, Vyacheslav Yu. Nikulin, Shamil R. Abdullin

**Affiliations:** Federal Scientific Center of the East Asia Terrestrial Biodiversity, Far Eastern Branch of the Russian Academy of Sciences, 159, 100-Letia Vladivostoka Prospect, Vladivostok 690022, Russia; chara1989@yandex.ru (V.B.B.); artyrozz@mail.ru (A.Y.N.); nikulinvyacheslav@gmail.com (V.Y.N.)

**Keywords:** Bacillariophyceae, diatom, integrative taxonomy, *rbc*L gene, light and scanning electron microscopy

## Abstract

The naviculoid genus *Luticola* exhibits a high degree of morphological convergence, complicating species delimitation when based solely on traditional morphometrics. Here, we describe *Luticola edaphica* sp. nov., a new species isolated from the forest soils of Mount Sestra (Primorsky Territory, Russian Far East) using an integrative taxonomic approach (phylogenetic, morphological, ultrastructural, and life cycle data). Molecular phylogenetic analysis, based on the chloroplast *rbc*L gene, placed the new strain within the *Luticola* clade, showing the closest affinity to *L. tenera*. However, *L. edaphica* is distinguished from similar *Luticola* species by a unique combination of morphological traits (structure of the valvocopula, maximal valve length and width, position and number of striae in 10 µm, central area, and distal raphe ends). A comprehensive study of its life cycle revealed that *L. edaphica* is homothallic and capable of both cis- and trans-anisogamy, with the latter being reported for the genus for the first time.

## 1. Introduction

Diatoms are the largest group of algae, with generally accepted estimates of their diversity ranging from tens to hundreds of thousands of species [1,2]. According to their last classification, diatoms include 431 genera among 68 families, 44 orders, and 10 classes [2]. They inhabit different aquatic and non-aquatic ecosystems, including soils, playing an important role in nature and for humans [3]. Most diatoms are photoautotrophs, but some species can grow without light by switching to mixotrophy [4,5]. The species of this group are promising objects for industrial cultivation because they synthesize various biologically active substances such as carotenoids, fatty acids, phenolic compounds, polysaccharides, and proteins [6,7,8].

The genus *Luticola* D.G. Mann was distinguished from the genus *Navicula* Bory and differs from it by uniseriate striae with more-or-less round poroids on the valve face, the presence of a single stigma, filiform raphe with deflected, bent, or hooked central and terminal endings, as well as a longitudinal canal positioned between the valve wall [3]. Species of *Luticola* are widely distributed in various habitats (aerophytic, soil, freshwater, and brackish water). In 2013, Levkov et al. [9] revised the genus *Luticola* and described 91 taxa new to science. In total, their monograph contained descriptions of about 200 species. To date, according to the AlgaeBase [1], the genus *Luticola* includes 274 species that are widely distributed around the world [9,10,11,12,13,14,15,16,17,18,19,20,21]. In 2023, a new species, *Luticola tenera* Bagmet, Abdullin, A. Nikulin, V. Nikulin, and Gontcharov, was described using an integrative approach for the soils of the Russian Far East (Jewish Autonomous Region) [22].

Despite the constantly growing number of new taxa, there are few molecular genetic data on the members of *Luticola*, for example [20,22]. Most members of this genus were based on morphological and morphometric traits, while some features of the valve ultrastructure, in particular the girdle, were often ignored. It is often noted that species delimitation based on morphology alone can lead to errors in taxonomic identification. This issue is mainly due to considerable variability in valve shape and size during the life cycle and the frequent presence of teratological forms [3,20,23,24,25,26]. Therefore, it is important to record any changes in the morphological and morphometric characteristics of the taxon during ontogeny, which cannot be achieved without observing the sexual process. This aspect has not been practically studied in the genus *Luticola*. So far, the sexual process has only been examined in six species [20,22,23,27].

During a study of algal diversity in soils in the Russian Far East (Primorsky Territory), a clone of a naviculoid diatom was isolated and analyzed using an integrative approach (molecular phylogenetics, morphological data, and the examination of life cycle). Based on the data obtained, we described this clone as new species of *Luticola*.

## 2. Results

### 2.1. Taxonomic Analysis

**Figure 1:**
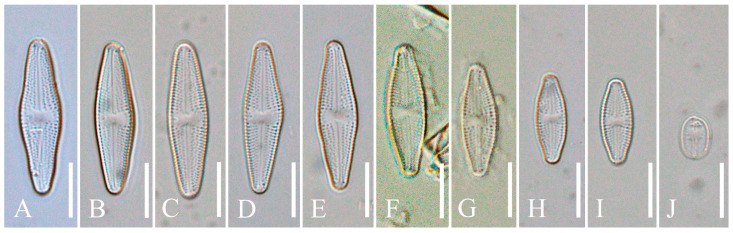
*Luticola edaphica* sp. nov. (**A**–**J**). Light microscopy (LM). Morphological changes in the size and shape of the valves during the life cycle. Scale bar: 10 μm.

**Figure 2:**
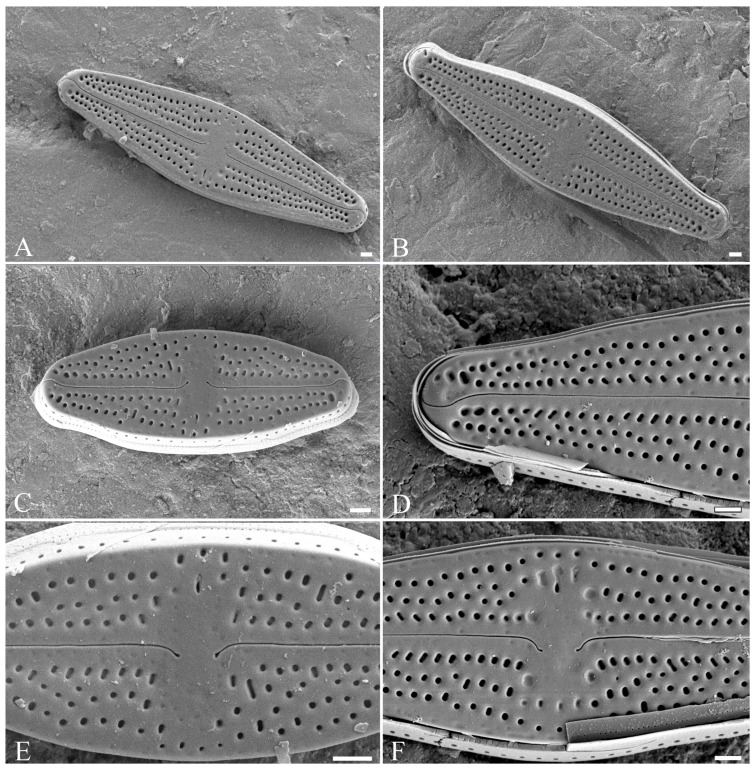
*Luticola edaphica* sp. nov. Scanning electron microscopy (SEM), external view (**A**–**F**): (**A**–**C**) general view of the valve with different shapes of ends; (**D**) distal raphe end; (**E**) small valve, central area with proximal raphe ends; (**F**) large valve, central area with proximal raphe ends. Scale bar: 1 μm.

**Figure 3:**
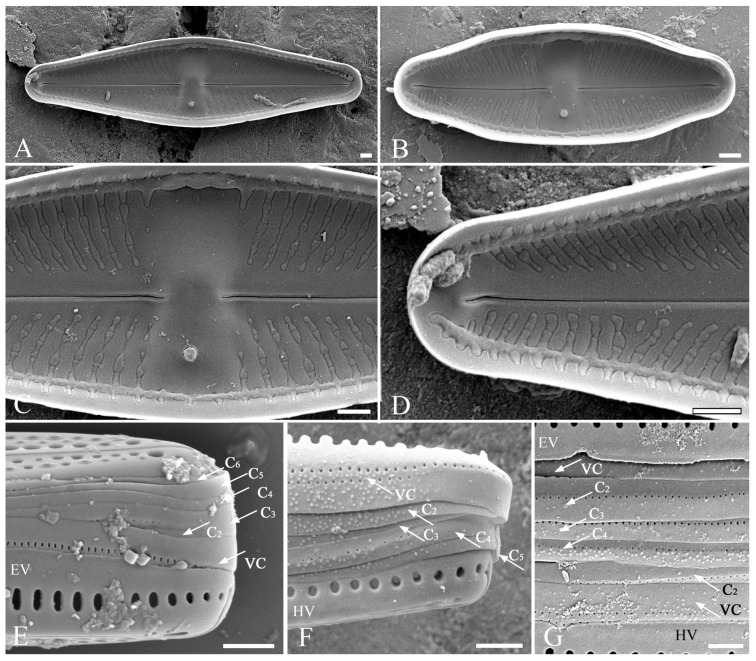
*Luticola edaphica* sp. nov. SEM, internal view. (**A**–**D**): (**A**) general view of large valve; (**B**) general view of small valve; (**C**) central area with proximal raphe ends and stigma; (**D**) distal raphe end terminated helictoglossa. (**E**–**G**) structure of girdle; (**E**) structure of the epicingulum (VC—valvocopula; C_2_–C_6_—copulae) and epivalva (EV); (**F**) detailed structure of valvocopula and copulae; (**G**) structure of the epitheca and part of the hypotheca, consisting of the hypovalva (HV) and two copulae. Scale bar: 1 µm.

*Luticola edaphica* V.B.Bagmet, A.Yu.Nikulin, V.Yu.Nikulin & Sh.R.Abdullin sp. nov. (Class Bacillariophyceae, Order Naviculales) is shown in Figure 1A–J, Figure 2A–F, and Figure 3A–G.

Holotype: Herbarium specimen accession number VLA-CA-1884, a dried biomass of a clone deposited in the Herbarium of the Federal Scientific Center of East Asian Terrestrial Biodiversity, Vladivostok, Russia (VLA). Gene sequence: DNA sequence obtained from a clone of *Luticola edaphica* was deposited in the GenBank under accession number PV930535.

Type locality: RUSSIA. Primorsky Territory. Nakhodka: Slopes of Mount Sestra, in the vicinity of the town (42°49′19.6″ N, 132°59′41.0″ E), on soil of temperate broadleaf forest.

Etymology: The species epithet “edaphica” is derived from the Greek “edaphos”, (=soil), named after its habitat—soil.

Distribution: Currently, it is only known from the slopes of Mount Sestra (Primorsky Territory, Russia).

Comments: It differs from similar *Luticola* species by the combination of morphological traits (structure of valvocopula, maximal valve length and width, position and number of striae in 10 µm, central area, proximal and distal raphe ends, and present of “ghost areolae”). It is distinct from most genetically related *L. tenera* in valve length and width, position and number of striae in 10 µm, central area, distal raphe ends, wavy edge of the valvocopula, differences in the chloroplast *rbc*L gene sequence, and the presence of trans-anisogamy.

Description: LM (Figure 1A–J). Cells are solitary. Valves from rhombic-lanceolate, lanceolate, and elliptic-lanceolate (Figure 1A–I) with broadly rounded (Figure 1A–D) or slightly protracted ends (Figure 1E–I) to elliptical with broadly rounded ends (Figure 1J). Valve dimensions (*n* = 40): length 8.3–29.5 μm; width 4.5–8.5 μm. Striae weakly radiate throughout, clearly visible in LM (Figure 1A–I), 14–22 in 10 μm. Central area wide, transversally elliptic or bow-tie-shaped (Figure 1A–I). Axial area narrow, linear, and slightly expanded in the central part (Figure 1A–I). Raphe straight, filiformis (Figure 1A–I). Proximal raphe ends poorly visible.

Scanning electron microscopy (SEM), external view (Figure 2A–F). Axial area narrow, slightly expanded to the valve center (Figure 2A–C). Central area transversally elliptic, covered with “ghost areolae”, making it slightly asymmetrical, and bordered on each margin by 3–4 isolated rounded areolae (Figure 2E,F). On the secondary part of the valve, there is one elongated slit-like stigma (Figure 2A–C,E,F). The striae weakly radiate, consisting of 2–4 round and elliptical areolae (Figure 2A–C). Raphe straight (Figure 2A–C). In small valves, proximal raphe ends are weakly asymmetrical, deflected opposite to the stigma (Figure 2E). In large valves, proximal raphe ends are weakly asymmetrical, deflected opposite to the stigma and hooked (Figure 2F). Distal raphe ends are hooked, first deflected towards the same side as the proximal ends, and then hooked towards the opposite side, extending onto the valve mantle (Figure 2D).

SEM, internal view (Figure 3A–D). Distal raphe ends branches terminating with small helictoglossae (Figure 3D). Proximal raphe ends straight (Figure 3C). Isolated pore single, round, close to the valve margin (Figure 3A–C). Areolae occluded by hymens, forming continuous strip across the valve (Figure 3A–D). Striae weakly radiate, 14–22 in 10 µm, composed of 2–4 rounded areolae. Marginal channel located on valve face/mantle junction, occluded with hymens (Figure 3A–D).

SEM, girdle (Figure 3E–G). Epivalve and hypovalve with a single row of rounded (Figure 3F) or elongated (Figure 3E) areolae. A mature epicingulum consists of 4–6 copulae (Figure 3E–G), each bearing a number of rounded areolae of the same morphological structure, 44–50 in 10 µm. One edge of the valvocopula is wavy (Figure 3F), while the other copulae have smooth edges (Figure 3F,G). These characters are stable across different individuals and stages of the life cycle. The valvocopula is perforated by two rows of pores (Figure 3F,G), whereas the other copulae have only one row of pores (Figure 3F,G). Valvocopula (VC) is the widest copula, other copulae are approximately equal in width. The hypocingulum has a similar structure (Figure 3G).

No teratological forms were observed.

### 2.2. Phylogenetic Analyses

**Figure 4:**
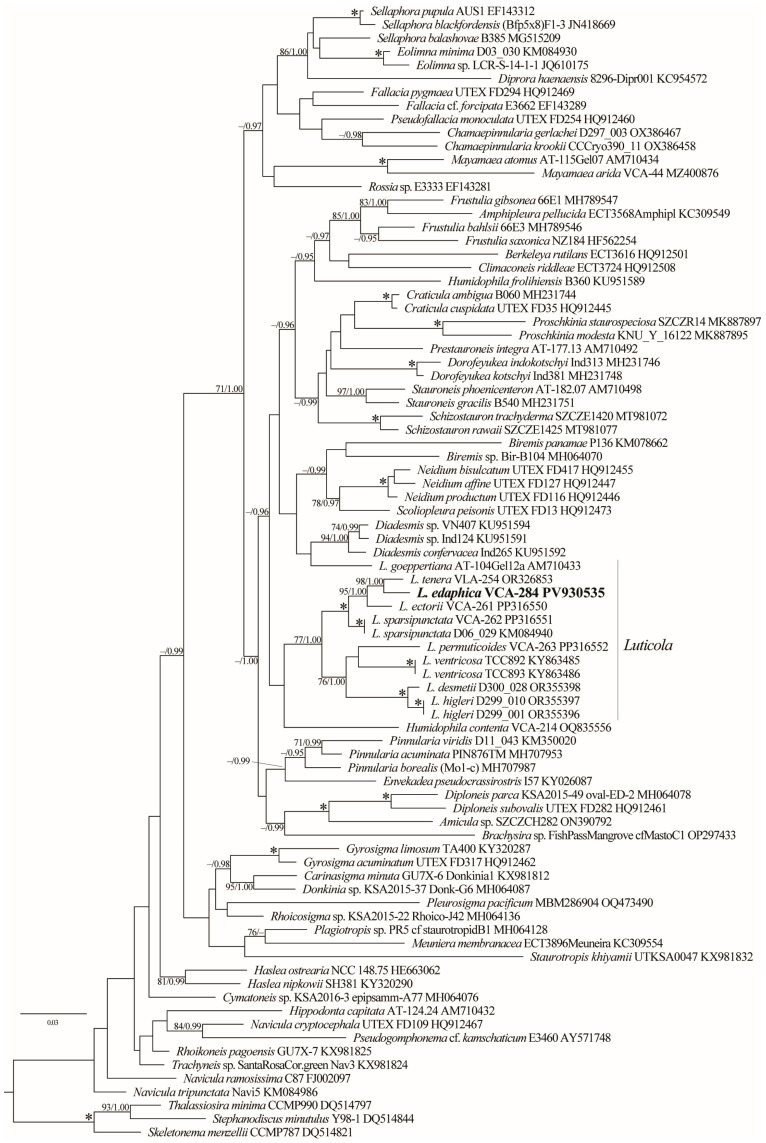
ML phylogenetic tree of the Naviculales (GTR + I + G model) showing the position of the new clone (VCA-284) based on partial *rbc*L gene sequence data (84 sequences, 1434 aligned positions). Support values [(BP) ≥ 70% and (PP) ≥ 0.95: ML/BI] are provided above/below the branches. The new clone is shown in bold. Nodes with 100% BP and 1.00 PP are marked with an asterisk. Scale bar: substitutions per nucleotide position.

The phylogenetic analysis based on 84 *rbc*L sequences revealed the paraphyletic nature of the genus *Luticola* with *L. goeppertiana* (Bleisch) Mann ex Rarick, Wu, Lee & Edlund, which was placed as a sister group to a strongly supported clade (94/1.00) containing species of the genus *Diadesmis* Kützing (Diadesmidaceae) (Figure 4). *Luticola s.s.* was divided into two distinct lineages. The first lineage (–/1.00) comprised the majority of *Luticola* accessions available in GenBank and was further subdivided into two clades. The first clade, exhibiting robust support (100/1.00), included *L. ectorii*, *L. edaphica*, *L. sparsipunctata*, and *L. tenera* with strong support, whereas the second clade, supported only in BI (–/1.00), consisted of *L. desmetii*, *L. higleri*, *L. permuticoides*, and *L. ventricosa*. The newly obtained sequence of *L. edaphica* showed affinity (98/1.00) to *L. tenera* (VCA–254).

### 2.3. Sexual Reproduction

Homothallic reproduction was observed in the monoclonal culture of *L. edaphica* with two types: cis- and trans-anisogamy.

**Figure 5:**
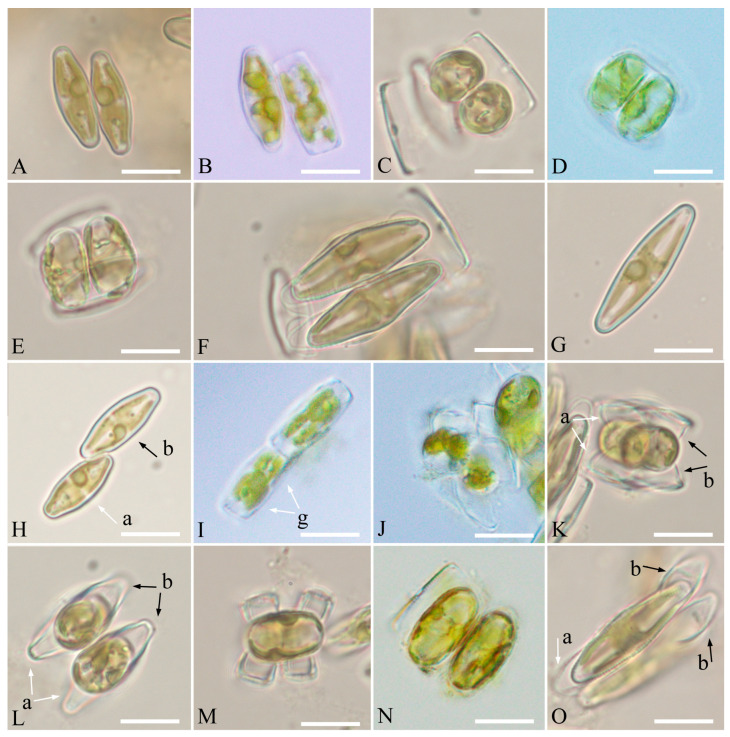
Sexual reproduction of *Luticola edaphica* sp. nov., LM: (**A**–**G**) cis-anisogamy: (**A**) pairing cells; (**B**) gametogenesis; (**C**) formation of zygotes; (**D**,**E**) growing auxospores; (**F**) initial cells leave the perizonium; (**G**) initial cell. (**H**–**O**) trans-anisogamy (a—parent 1, white arrow, b—parent 2, black arrow): (**H**) pairing cells; (**I**) gametogenesis (gametes (g) are indicated by white arrows); (**J**) syngamy and formation of zygotes; (**K**) zygotes; (**L**–**N**) growing auxospores; (**O**) mature auxospores. Scale bar: 10 µm.

Cis-anisogamy (Figure 5A–G): The process proceeds as in *L. dismutica* [27], *L. ectorii* [20], *L. poulickovae* [23], *L. sparsipunctata* [20], and *L. tenera* [22]. It is designated as IA2a, according to Geitler [28].

Trans-anisogamy (Figure 5H–O): Two cells connect apically, forming a gametangial pair (Figure 5H). The cells become stationary after adhesion, and meiosis begins. It is followed by cytokinesis, which results in the formation of two gametangia. The protoplast of each gametangium transapically divides, forming two morphologically identical gametes (Figure 5I). One motile gamete and one immotile gamete are formed in each gametangium. The gametes are rounded and rearranged, and the motile gametes move to the apical part of the valve, which connects with another gametangium. A motile gamete from one gametangium moves to another gametangium containing an immotile gamete. As a result of syngamy, two spherical zygotes are formed (Figure 5J,K). The zygotes bipolarly expand and elongate parallel to the valves of the parent cells (Figure 5L–N), turning into auxospores (Figure 5O). This type of sexual reproduction could be classified as trans-anisogamy. It is designated as IA1a, according to Geitler [28].

**Figure 6:**
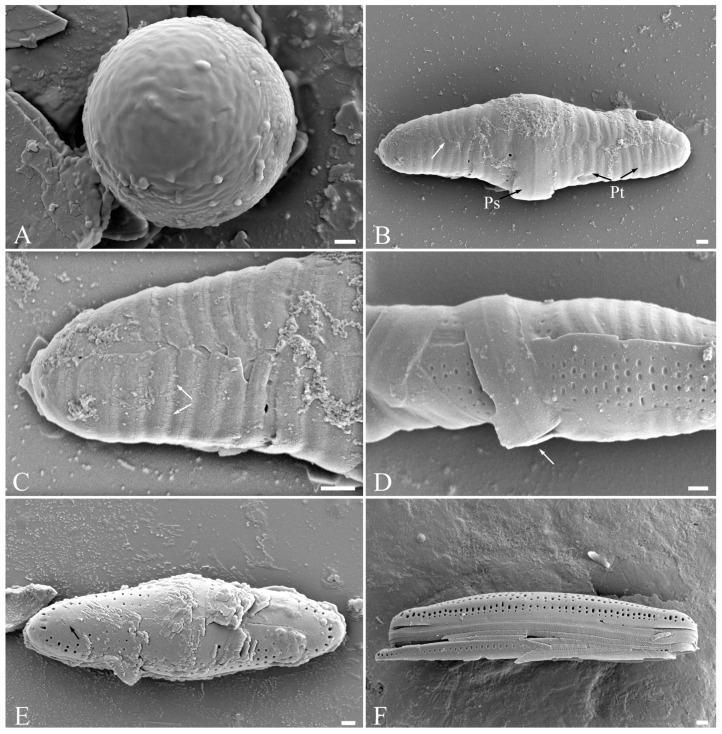
*Luticola edaphica* sp. nov. SEM: (**A**) incunabula; (**B**–**D**) structure of the perizonium; (**B**) primary (Ps) and secondary (Pt) perizonium bands, the white arrow indicates the line of rupture of the secondary perizonium bands; (**C**) secondary perizonium bands with fringed edges (arrows); (**D**) primary perizonium band with a gap (arrow); (**E**) initial cell, the black arrow indicates the distal raphe end on the front part of the valve; (**F**) initial cell, girdle view.

The zygote is covered with incunabula (Figure 6A), which, expanding transversely, turns into a perizonium (Figure 6B–D). The auxospore perizonium is formed by transverse elements (Figure 6B–D). The primary perizonium band is the widest and has a break (Figure 6B,D). The secondary bands are narrower, with a fringed edge, and have a break line on the side of the perizonium (Figure 6B,C). The initial cell is formed inside of the fully grown auxospore that morphologically differs from vegetative cells: its shape is more rounded, there is no clear distinction between the external valve and the mantle, and the distal raphes end on the surface of the valve (Figure 6E,F). The number of areolae forming striae in the central area is 4–5 in the auxospores and is 2–4 in the vegetative cells. A fully formed initial cell emerges by breaking the perizonium and begins active vegetative division.

## 3. Discussion

Levkov et al. [9] distributed 200 European *Luticola* species between 17 artificial groups (A–Q) based on a combination of eight morphological traits (length and width, shape, apices, stria density in 10 μm, central area, axial area, distal raphe ends, and proximal raphe ends). The morphological features of *L. edaphica* at different stages of the life cycle fit the characteristics of some species from groups C, D, and O. Consequently, a comparative morphological analysis was conducted between the investigated clone and members of these groups (Table S1). Of these, seven species are the most similar to *L. edaphica*: *L. falknerorum* Metzeltin & Lange-Bertalot; *L. frickei* Levkov, Metzeltin & A.Pavlov; *L. fuhrmannii* Metzeltin & Levkov; *L. gesierichiae* Levkov, Metzeltin & A.Pavlov; *L. imbricatiformis* Levkov, Metzeltin & A.Pavlov; *L. intermedia* (Hustedt) Levkov, Metzeltin & A.Pavlov; and *L. nana* Levkov, Metzeltin & A.Pavlov. We also compared the morphology of the new species with genetically related species: *L. ectorii*, *L. sparsipunctata*, and *L. tenera* (Figure 4; Table S1).

Among the analyzed species, the shortest valve length was observed in *L. edaphica* and *L. sparsipunctata* and the minimum valve width was observed in the new species, *L. sparsipunctata*, and *L. frickei* (Table S1). The valves of *L. sparsipunctata* and *L. tenera*, as well as *L. edaphica*, have slightly protracted or broadly rounded ends. In position and number of striae in 10 µm, the new species is closest to *L. sparsipunctata*; however, the striae in the latter consist of a smaller number of areolae. In the central area, the new taxon is similar to *L. nana*, but the axial area is the same in many of the analyzed species: *L. ectorii*, *L. falknerorum*, *L. fuhrmannii*, *L. imbricatiformis*, *L. nana*, *L. sparsipunctata*, and *L. tenera*, including *L. edaphica*. The proximal raphe ends in large cells of the new species are similar to those of *L. ectorii* and *L. tenera*, while in small cells they differ from those of other species. The distal raphe ends of *L. edaphica* are the same as in *L. imbricatiformis*, *L. fuhrmannii*, *L. intermedia*, *L. frickei*, and *L. gesierichiae*. “Ghost areolae” are observed in the new taxon, as well as in the species *L. falknerorum*, *L. ectorii*, and *L. tenera*. The girdle of *L. edaphica* is most similar to the girdle of *L. ectorii*, *L. sparsipunctata*, and *L. tenera*, but the main difference between the new species and these three taxa is the presence of a wavy edge of the valvocopula. (Table S1). This feature was previously found in the species *L. georgzizkae* Witkowski, Lange-Bertalot, M.Rybak & Peszek [17] and *L. scardica* Levkov, Metzeltin & A.Pavlov [9], but these species differed from *L. edaphica* in most morphological characteristics. According to our previously proposed classification of *Luticola* species by girdle [20], the new species belongs to the third group of perforation. This group also includes the species *L. asiatica* Lokhande, Lowe, Kociolek & Karthick, *L. ectorii*, *L. georgzizkae*, *L. ivetana* Chattová & Van de Vijver, *L. rapanuiensis* Rybak, Peszek, Witkowski & Lange-Bertalot, *L. rojkoviensis*, and *L. sparsipunctata*. The closest species to *L. edaphica* in terms of habitat was *L. tenera*, although it was found in waterlogged floodplain soil, while our species was found in forest soil (Table S1). Thus, according to morphological characteristics, *L. edaphica* is most similar to *L. sparsipunctata* and *L. tenera* (which is genetically closest to the new species), but differs from the first in maximal valve length and width, central area, proximal and distal raphe ends, absence of “ghost areolae”, wavy edge of the valvocopula, habitat, and from the second in valve length and width, position and number of striae in 10 µm, central area, distal raphe ends, and wavy edge of the valvocopula. Consequently, the combination of morphological features (structure of valvocopula, maximal valve length and width, central area, etc.) differentiates our taxon from morphologically similar and genetically close *Luticola* species.

As we previously described for *L. tenera* [22], features such as the shape and ends of the valves and the number of areolae forming striae vary during the life cycle of *L. edaphica*. A similar trend was described in different genera of diatoms inhabiting soils [29]. Therefore, it is once again confirmed that these morphological traits are not constant in *Luticola* and cannot be used as a species-specific character.

The resulting phylogenetic tree further confirms the polyphyletic nature of the genus *Luticola*, as previously suggested by Bagmet et al. [20,22]. Most representatives of *Luticola* are grouped into a major lineage, which itself is composed of at least two distinct subclades. Notably, *L. goeppertiana* is placed outside the main *Luticola* lineage and is resolved as a sister taxon to a well-supported clade of *Diadesmis* species. This result contrasts with the findings of Kulikovskiy et al. [30], who recovered *Luticola* as monophyletic based on SSU rDNA + *rbc*L data. The taxon sampling in that analysis is also limited and does not include many recently described or molecularly characterized species. As more sequence data become available, particularly from type or reliably identified strains, a multi-gene analysis may help to reconcile these conflicting topologies and provide a more robust framework for understanding evolutionary relationships within the genus.

All species of the genus *Luticola* with studied sexual reproduction, as well as *L. edaphica*, are homothallic. In five species (*L. ectorii* [20], *L. dismutica* [27], *L. poulickovae* [23], *L. sparsipunctata* [20], and *L. tenera* [22]), behavioral cis-anisogamy (IA2a according to Geitler [28]) was revealed. In addition, isogamy (IC according to Geitler [28]) was noted in *L. tenera* [22] and another type of isogamy (IB2b according to Geitler [28]) was found in *L. permuticoides* [20]. In *L. tenera*, for example, a transition is observed from the normal type of sexual process, in which two gametes are formed in the gametangial cell, to a reduced one, in which two gametes are also formed in the gametangial cell, but one then dies [22]. In *L. permuticoides*, only one gamete is always formed in the gametangium [20]. In the new species *L. edaphica*, two types of sexual reproduction were also noted: cis- and trans-anisogamy. Moreover, trans-anisogamy has been observed in species of the genus for the first time. Thus, all studied taxa of the genus *Luticola* have a wide variety of sexual process types, but all these species are homothallic. Further research is needed to clarify these features.

The integration of morphological and ultrastructural data with molecular phylogenetics and analysis of life cycle in this study demonstrates that *L. edaphica* represents a novel species. This case further underlines the need for an integrative approach to the characterization of *Luticola* taxa from understudied regions and habitats, particularly terrestrial ecosystems, where cryptic diversity is likely to be high. Given the unresolved boundaries and the poor representation of many taxa in molecular databases, it is likely that the true diversity and evolutionary history of *Luticola* remain underestimated. Future studies employing multigene datasets and broader taxon sampling are essential to resolve the phylogenetic relationships within this complex and diverse genus.

## 4. Materials and Methods

### 4.1. Sampling and Culture Conditions

A sample of soil was collected in a broadleaf forest on the slopes of Mount Sestra, in the vicinity of the town of Nakhodka (Primorsky Territory, Russia; 42°49′19.6″ N, 132°59′41.0″ E) on 20 June 2022 using standard methods [31]. A clone of naviculoid diatom was isolated via the micro-pipette method [32] and incubated in 40 mm Petri dishes with liquid nutrient medium Dm [33] under the following conditions: 20–22 °C, photon fluence 17.9–21.4 μmol photons·m^−2^ s^−1^, and 16:8 h light:dark cycle. The clone was kept in the culture collection of the Laboratory of Botany in the Federal Scientific Center of East Asian Terrestrial Biodiversity, Russian Federation (clone number VCA–284), and its dried biomass was deposited in the Herbarium of the Federal Scientific Center of East Asian Terrestrial Biodiversity, Russia (herbarium specimen number VLA-CA-1884).

### 4.2. Microscopy

The morphology and morphometrics of the cells were studied using an Olympus BX53 light microscope (LM) (Olympus Corporation, Tokyo, Japan) equipped with Nomarski DIC optics and an Olympus DP27 digital camera (Olympus Corporation, Tokyo, Japan), as well as a Merlin scanning electron microscope (SEM) (Carl Zeiss, Jena, Germany). Frustules were cleaned via oxidation with hydrogen peroxide (Dalnevostochnaya laboratoriya, Vladivostok, Russia), rinsed several times with distilled water, and mounted in a Pleurax medium. The material was dried onto brass stubs and coated with a gold–palladium (Au–Pd, 6:4) alloy for SEM. The morphometric data were analyzed using the software package Statistica 10.0 and Microsoft Office Excel 2007 (https://www.microsoft.com/).

### 4.3. Mating Experiments

Sexual reproduction in our clone was observed during cultivation under the conditions described above. Mixed cells were examined daily with an inverted light microscope CK30-F200 (Olympus Corporation, Tokyo, Japan) for three weeks in December 2022–January 2023. Living cells, auxosporulation, and the stages of sexual reproduction were observed and described using LM following the methods described by Poulíčková and Mann [34] and Poulíčková et al. [35].

### 4.4. DNA Extraction, Amplification and Sequencing

For DNA analysis, the culture was harvested during the exponential growth phase and concentrated via centrifugation. Subsequently, DNA extraction, PCR amplification, and sequencing of the plastid-encoded *rbc*L gene were conducted according to the methodology outlined by Bagmet et al. [22]. The PCR products were sequenced in both directions at the Instrumental Centre of Biotechnology and Gene Engineering of FSCEATB FEB RAS. Sequences were assembled with the Staden Package v.1.4 [36]. The contig sequence covering the partial *rbc*L gene was deposited in GenBank under accession number PV930535.

### 4.5. Alignment and Datasets

For phylogenetic analyses, an alignment including 84 taxa of the order Naviculales Bessey (1434 bp) was constructed based on the dataset presented by Bagmet et al. [20]. Three species of centric diatoms were selected as the outgroup. The sequences were aligned using the SeaView program [37] with manual corrections.

### 4.6. Phylogenetic Analysis

Maximum likelihood (ML) analysis was carried out using PAUP 4.0b10 [38], and Bayesian inference (BI) was performed using MrBayes 3.1.2 [39]. The GTR + I + G nucleotide substitution model was selected as the optimal for both methods according to the Akaike Information Criterion (AIC; [40]) in jModelTest 2.1.1 [41]. The ML and BI analyses were conducted according to Bagmet et al. [22]. The ML-based rapid bootstrap analysis was performed using RAxML-NG (https://github.com/amkozlov/raxml-ng; [42]; accessed on 8 July 2025). The robustness of the ML trees was estimated by bootstrap percentages (BP) and posterior probabilities (PP) in BI. BP < 70% and PP < 0.95 were not considered.

## 5. Conclusions

*Luticola edaphica* from the Russian Far East was described as a new species using an integrative taxonomic approach. We characterized morphology, phylogenetic data and sexual reproduction in the species during its life cycle and showed that it is distinguished from similar *Luticola* species. Trans-anisogamy has being reported for the genus for the first time.

## Data Availability

The data presented in this study are available on request from the corresponding author. In addition, the data that support the findings of this study are openly available in GenBank.

## Supplementary Materials

The following supporting information can be downloaded at: https://www.mdpi.com/article/10.3390/plants15060897/s1, Table S1: Comparative analysis of morphological and morphometric parameters in *Luticola edaphica* sp. nov. and morphologically similar/genetically related species.
