# Supplementary material for: Luticola edaphica sp. nov. (Diadesmidaceae, Naviculales) from the Soil of the Russian Far East (Primorsky Territory, Russia)
Source: Plants (Basel). 2026 Mar 13;15(6):897. doi: 10.3390/plants15060897 (PMC13029660; doi:10.3390/plants15060897)
Supplement: Supplementary file 1 [file plants-15-00897-s001.zip › Table S1.pdf]

Table S1. Comparative analysis of morphological and morphometric parameters in *Luticola edaphica sp. nov.* and morphologically similar / genetically related species.

Character	Morphologically similar species (groups according Levkov et al., 2013)							Genetically related species			***L. edaphica sp. nov.***
	C		D			O					
	L. falknerorum	***L. imbricatiformis***	***L. fuhrmannii***	***L. intermedia***	***L. nana***	***L. frickei***	***L. gesierichiae***	***L. ectorii***	***L. sparsipunctata***	***L. tenera***	
Length, µm	15.5–46.0	12.0–34.0	18.0–34.0	10.0–29.0	11.0–14.0	12.0–31.0	16.0–39.0	10.0–37.1	5.0–28.0	15.7–35.0	8.3–29.5
Width, µm	7.5–10.0	5.5–7.0	7.5–8.5	5.5–8.5	5.5–6.0	4.5–7.5	6.0–10.5	5.5–9.5	4.1–9.1	6.6–11.0	4.5–8.5
Valve shape	linear-lanceolate to elliptic-lanceolate	linear-lanceolate to elliptic	broadly lanceolate to rhombic-elliptic, asymmetric (dorsiventral)	asymmetric, lanceolate to rhombic-elliptic	rhombic-lanceolate to elliptic-lanceolate	linear-lanceolate to linear	lanceolate to linear-lanceolate	rhombic to rhombic-elliptical	rhombic-lanceolate, lanceolate to elliptical	rhombic-lanceolate, lanceolate to elliptic-lanceolate	rhombic-lanceolate, lanceolate, elliptic-lanceolate to elliptical
Apices	broadly rounded	broadly rounded, not protracted	broadly rounded, not protracted	broadly rounded, not protracted	weakly rostrate and broadly rounded	slightly protracted, rounded to not protracted, narrowly rounded	slightly protracted and rounded	truncated or broadly rounded	slightly protracted or broadly rounded	broadly rounded, non-protracted to slightly protracted	broadly rounded or slightly protracted
Striae	radiate throughout, 21–24 in 10 µm, composed of 5–6 round to elongated areolae	radiate in mid-valve, strongly radiate towards apices, 15–19 in 10 µm, composed of 3–4 round to elongated areolae	radiate near mid-valve, strongly radiate towards apices, 22–24 in 10 µm, composed of 3–4 round to elongated areolae	radiate near mid-valve, strongly radiate towards apices, 22–24 in 10 µm, composed of 4–5 round to elongated areolae	radiate throughout, 22–25 in 10 µm, composed of 3–4 round to elongated areolae	radiate near mid-valve, strongly radiate towards apices, 18–21 in 10 µm, composed of 4–5 round to elongated areolae	radiate near mid-valve, strongly radiate towards apices, 15–18 in 10 µm, composed of 3–4 round to elongated areolae	radiate, 15–21 in 10 µm, composed of 2–5 round areolae	weakly radiate, 15–22 in 10 µm, composed of 2 (rarely 3) round to elliptical areolae	weakly radiate, 17–23 in 10 µm, composed of 2–6 round areolae	weakly radiate throughout, 14–22 in 10 µm, composed of 2–4 round and elliptical areolae
Central area	broad, elliptic to transversally elongated, bordered on each margin by 2–5 isolated round areolae	wide, transversally elliptic, to wedge–shaped, bordered on each margin by 3–5 isolated round areolae	asymmetrical, rectangular to bow-tie-shaped, bordered on each margin by 3–4 isolated round areolae	wide, asymmetrical, wedge-shaped, to bow-tie-shaped, bordered on each margin by 4–5 isolated	wide, transversally elliptic to bow-tie-shaped, bordered on each margin by 3–4 round areolae	wide, weakly asymmetrical, rectangular to bow-tie-shaped, bordered on each margin by 2–3 round areolae	wide, asymmetrical, rectangular to wedge-shaped, bordered on each margin by 2–4 isolated round areolae	wide, rectangular or bow-tie-shaped, bordered on each margin by 3–7 round areolae	wide, bordered by 2–5 round areolae on both sides	weakly asymmetrical, rectangular, bordered on each margin by 3–5 isolated round areolae	wide, transversally elliptic or bow-tie-shaped, slightly asymmetrical, bordered on each margin by 3–4

				round areolae							isolated rounded areolae
Axial area	narrow, linear, widened near central area	narrow, linear, slightly expanded near central area	narrow, linear, slightly expanded near central area	narrow, linear throughout	narrow, almost linear, slightly expanded near central area	narrow, linear, weakly expanded near central area	linear, weakly expanded near central area	narrow, linear, slightly expanded near central area	linear, slightly expanded in the central part of the valve	linear, narrow, expanded in the central part of the valve	narrow, linear, and slightly expanded in the central area
Proximal raphe ends	hooked or just deflected opposite to stigma	hook-shaped, deflected to side opposite to stigma and expanded into central pores	long, deflected to side opposite to stigma and expanded into central pores	deflected to side opposite to stigma and expanded into central pores	deflected opposite to stigma and expanded into central pores	close standing, hooked on opposite side of stigma	deflected opposite of stigma	weakly asymmetrical (different angle of inclination and depth of the bend, in the form of a hook) or deflected to the side opposite to stigma	deflected to the side opposite to the stigma	weakly asymmetrical, hooked or just deflected opposite to stigma	in small valves – weakly asymmetrical, deflected opposite to the stigma. In large valves – weakly asymmetrical, hooked or deflected opposite to the stigma
Distal raphe ends	hooked, first deflected towards same side as proximal ends, then hooked towards opposite side	hooked, first deflected towards same side as proximal ends, then hooked towards opposite side, extending onto valve mantle	hooked, first deflected towards same side as proximal ends, then hooked towards opposite side, continuing onto valve mantle	hooked, first deflected towards same side as proximal ends, then hooked towards opposite side, continuing onto valve mantle, terminating shortly before valve edge	short, linear or slightly deflected towards same side as proximal ends	hooked, first deflected towards same side as proximal ends, then hooked towards opposite side, continuing onto valve mantle	hooked, first deflected towards same side as proximal ends, then hooked towards opposite side, continuing onto valve mantle	first deflect in the same direction as proximal ends and then become hooked	first deflect to the same side as the proximal ends, and then hook-like bend in the opposite direction	hooked, first deflected towards same side as proximal ends, then hooked towards opposite side	hooked, first deflected towards same side as the proximal ends and then hooked towards opposite side, extending onto the valve mantle
Ghost areolae	present	nd	nd	nd	nd	nd	nd	present	absent	present	present
Girdle	mantle with single row of elongated areolae	nd	mantle with single row of round to elliptic	mantle with single row of round areolae	nd	mantle with single row of round areolae; each	mantle with single row of large, elliptic areolae; each	mantle with single row of elliptical areolae;	mantle with single row of rounded areolae;	mantle with single row of elongated areolae; a	mantle with single row of rounded or elongated

			areolae			girdle band with two rows of small poroids	girdle band with two rows of small poroids	epicingulum consists of six copulae, of which the valvocopula is the widest copula, and it is perforated by two rows of rounded areolae; last copulae are perforated by one row of rounded pores	epicingulum of large valves consists of six copulae, small valves – of nine copulae; valvocopula is perforated by two rows of round areolae; last copulae are perforated by one row of round pores of the same structure	mature cingulum consists of five copulae, each bearing a number of rounded areolae of the same morphological structure, 55–60 in 10 µm; valvocopula is the widest copula, the second and the third copulae, the fourth and the fifth copulae are approximately equal in width	areolae; a mature epicingulum consist of 4–6 copulae, each bearing a number of rounded areolae of the same morphological structure, 44–50 in 10 µm; one edge of valvocopula is wavy, while the other copulae have smooth edges; valvocopula is perforated by two rows of pores, whereas the other copulae have only one row of pores; valvocopula is the widest copula, other copulae are approximately equal in width
Habitat	nd	stone (epilithic)	nd	electrolyte rich waters	nd	nd	nd	lake; epiphythic on freshwater macroalgae; epilithic on architectural structure in urban ecosystem	freshwater; epiphythic on deciduous trees; epilithic on architectural structure in urban ecosystem	waterlogged soil	soil
Locality	tropical, the Pazaino,	Lake Tanganyica,	tropical, Amany Ost-	tropical / subtropical	Rio Lauca, 4000 m a.s.l.,	Tanzania	Tanzania	Brazil, China, Java, Laos,	Europe, Russia,	State Nature Reserve	Mount Sestra (Primorsky

	Brook-sand (Colombia) and South Africa	M'Pulungu, Ile Niamcolo.	Usambara-gebirge Korogwetal, Tanga, Tanzania; Brazil; Uruguay	regions	Chile			Russia, Vietnam		«Bastak», Jewish Autonomous Region, Russia	Territory, Russia)
Source	Levkov et al., 2013	Levkov et al., 2013	Levkov et al., 2013	Levkov et al., 2013	Levkov et al., 2013	Levkov et al., 2013	Levkov et al., 2013	Levkov et al., 2013; Glushchenko, Kulikovskiy, 2015; Bagmet et al., 2024	Levkov et al., 2013; Rybak et al., 2023 Bagmet et al., 2024	Bagmet et al., 2023	this study

Notes: nd – no data.
